# In Vivo Quantification of White Matter Pathways in the Human Hippocampus

**DOI:** 10.1002/hbm.70417

**Published:** 2025-11-24

**Authors:** Melisa Gumus, Athanasios Bourganos, Michael L. Mack

**Affiliations:** ^1^ Department of Psychology University of Toronto Toronto Ontario Canada

**Keywords:** diffusion magnetic resonance imaging, hippocampal pathways, hippocampus, humans, white matter

## Abstract

The hippocampus is a key structure in cognition. Although much research has focused on defining the functions of its anatomically distinct subfields, the communication among these subfields within the hippocampal circuit, supported by white matter pathways, is theoretically key to emergent cognitive function. Yet, hippocampal white matter connections in humans have not been fully explored in vivo. By leveraging diffusion‐weighted imaging and a large healthy sample (*N* = 653), we developed a processing pipeline for in vivo quantification of human hippocampal pathways. We provided evidence for monosynaptic and trisynaptic pathway‐related connections in humans, supporting the described hippocampal circuit in ex vivo and animal studies. In addition to hemispheric and sex differences, the individual variability in hippocampal pathways was linked to cognitive abilities. Thus, in vivo characterization of human hippocampal pathways highlights the individual differences within these structures and paves the way for their implications in cognition, aging, and diseases.

## Graphical abstract

By leveraging diffusion‐weighted imaging and a large healthy sample (*N* = 653), we developed a processing pipeline for in vivo quantification of human hippocampal pathways. We provided evidence for hippocampal pathway‐related connections in humans, supporting the described hippocampal circuit in ex vivo and animal studies.

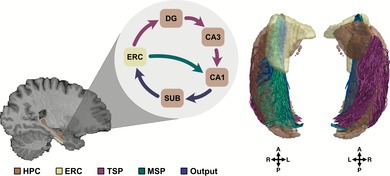

## 1 Introduction

The hippocampus is a critical brain region in supporting a wide range of cognitive functions including episodic memory, category learning, spatial navigation, and decision making (Eichenbaum 2004; Mack et al. 2018; Schapiro et al. 2017; Scoville and Milner 1957; Shohamy and Turk‐Browne 2013). With its extensive involvement in cognition, it is also implicated in a variety of medical conditions from neurodegenerative to psychiatric disorders (de Flores et al. 2015; Foo et al. 2017; Maruszak and Thuret 2014). Histological and cytoarchitectural evidence highlights anatomically distinct hippocampal subfields such as dentate gyrus (DG), cornu ammonis area 1 (CA1), CA2/3/4, and subiculum (SUB) as well as entorhinal cortex (ERC) as the major input/output region (Duvernoy et al. 2013). These structures are recruited during many cognitive functions (Aimone et al. 2011; Leutgeb et al. 2007; O'Reilly et al. 2014) as information travels within the hippocampal pathways connecting the subfields and ERC (Anand and Dhikav 2012). Yet, there is limited empirical in vivo characterization of human hippocampal white matter connections, which is fundamental to understanding the role of the hippocampus in cognition and its implications in disorders (Karat et al. 2023; Yassa et al. 2011; Yassa et al. 2010; Zeineh et al. 2001; Zeineh et al. 2017).

**FIGURE 1:**
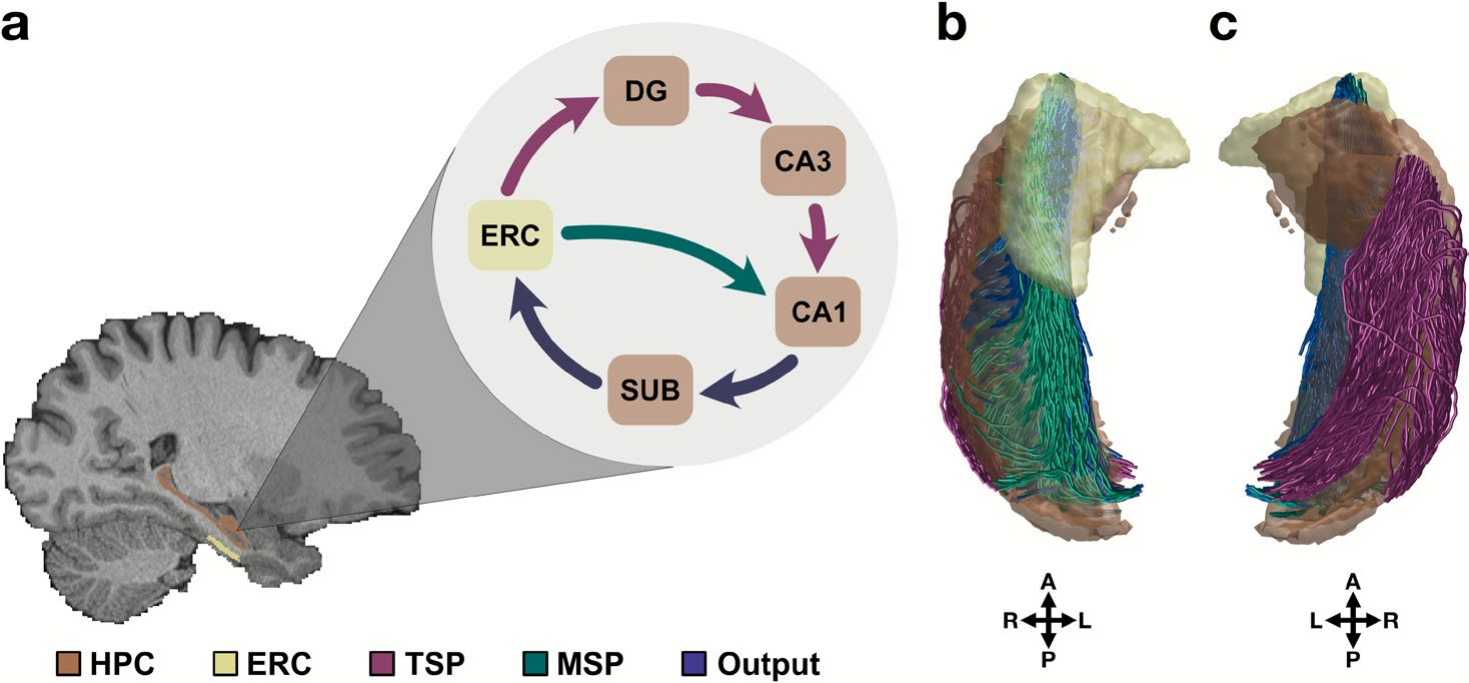
Theorized hippocampal circuit and in vivo quantification of its connections in humans. (a) Hippocampal circuit links hippocampal subfields and ERC. MSP connects ERC to CA1 while TSP spans across ERC, DG, CA3, and CA1. We refer to the CA1‐SUB and SUB‐ERC as output connections since they complete the hippocampal circuit. (b) Quantified TSP‐related connections in humans are visible from a superior view. (c) MSP‐related and output connections in humans are observed from an inferior view. Note: HPC = hippocampus.

Our knowledge regarding the hippocampal circuit is mostly inferred from animal studies (David and Pierre 2006; Patten et al. 2015), which motivates the theoretical framework for the flow of information within the hippocampal circuit (Figure 1a) (O'Reilly et al. 2014; Schapiro et al. 2017). There are two main pathways linking the hippocampal subfields and ERC: trisynaptic (TSP) and monosynaptic (MSP) (Patten et al. 2015). ERC is the main input of the hippocampal circuit, and thus of both pathways (David and Pierre 2006; Witter et al. 2017). As part of TSP, ERC projects its unidirectional connections onto DG through the perforant pathway (Patten et al. 2015; Witter et al. 2017). Unmyelinated mossy fibers then link DG to CA3, which then connects to CA1 via Schaffer collaterals. MSP, on the other hand, is composed only of ERC connections that directly project onto CA1 without stopping at DG (Yeckel and Berger 1995). To complete the hippocampal circuit, CA1 connects to SUB, which is then linked back to ERC (Patten et al. 2015). Besides ERC, SUB is also considered an output from the hippocampal circuit, delivering processed information to cortical and subcortical regions although its specific functions in cognition remain elusive (Roy et al. 2017). Post‐mortem work points to a high resemblance of the described hippocampal circuit in humans (Beaujoin et al. 2018). However, the challenge has been to investigate these structures in humans with an in vivo design.

Developments in magnetic resonance imaging (MRI) techniques within the last two decades enabled in vivo measurements of the human hippocampal subfields (DeKraker et al. 2021; Iglesias et al. 2015; Mueller et al. 2007; Van Leemput et al. 2009; Yushkevich et al. 2009). Extensive descriptions of the anatomical distinctions between the hippocampal subfields advanced research on their involvement in cognition (Córdova et al. 2019; Duncan et al. 2012; Suthana et al. 2015). For example, human CA1 integrates new experiences with the existing memories that have overlapping features (Schlichting et al. 2014) while CA3 and DG separate certain experiences and store them as distinct representations (Bakker et al. 2008; Lacy et al. 2011). One missing piece of the puzzle in the hippocampal circuit is understanding the role of hippocampal pathways connecting these subregions. Although the neuroanatomy of the hippocampal formation is largely consistent across species such as monkeys, rats, and humans, there exist substantial structural differences in the organization of its connections with itself and cortical areas (Clark and Squire 2013; David and Pierre 2006; Zeineh et al. 2017). Thus, in vivo approaches are key to characterizing the cognitive relevance of white matter pathways of the human hippocampal circuit.

Diffusion imaging is the dominant technique for in vivo evaluation of white matter connections in the human brain, revealing insights into their structure and the underlying cognitive processes (Le Bihan and Johansen‐Berg 2012). The particular imaging relies on Brownian motion of water molecules which diffuse more freely in the direction of fibers, allowing differentiation of white matter structures (Le Bihan and Johansen‐Berg 2012). Although the hippocampus is a relatively small region, novel diffusion processing methods have enabled researchers to investigate broader hippocampal connections with cortical and subcortical regions in humans (Dalton et al. 2022; Huang et al. 2021; Maller et al. 2019). Distinct cortical regions, including the medial temporal lobe, parietal and occipital regions, are preferentially connected along the anterior–posterior axis of the human hippocampus (Dalton et al. 2022). In vivo neuroimaging methods also infer measures of neuroplasticity within the human hippocampus such as remodeling of white matter connections during learning, even within short time intervals (Sagi et al. 2012). Such tools are also feasible in investigating aging‐related changes within the human hippocampal circuit. For example, white matter connections between the hippocampal subfields present reduced structural integrity in older adults (Elsaid et al. 2022) and the degradation of perforant connections relates to memory deficits (Yassa et al. 2011). Novel in vivo neuroimaging techniques and processing pipelines specific to the human hippocampus are critical in understanding the role of the hippocampal circuit in cognition. Thus, it is necessary to characterize in vivo hippocampal white matter connections in a large sample to establish a normative model of the human hippocampal circuit and the individual variability among its interconnections.

Although diffusion techniques are powerful in in vivo investigations, certain limitations need to be addressed (Schilling et al. 2019). The lack of sensitive processing methods and the difficulty with their validation and reproducibility could pose a challenge to characterizing the human hippocampal pathways. To increase transparency and reproducibility, we provide a detailed protocol and documentation on the processing pipeline for hippocampal white matter connections. The large sample size included in this study improves the generalizability of the results. There have also been limitations around image resolution and the complex microstructure of white matter pathways such as crossing fibers. However, innovative approaches in diffusion MRI allow for more sensitive and detailed examination of white matter connections in humans (Fan et al. 2016; McNab et al. 2013) with the ability to evaluate fiber orientations within a voxel and reconstruct tractography with high resolution (Basser et al. 2000; Wedeen et al. 2012). These developments allow for white matter connections to be estimated within the brain regions with complex structural organization, such as the hippocampus. Indeed, such algorithms previously revealed the hippocampal connections with the rest of the brain (Dalton et al. 2022; Huang et al. 2021). However, there has not been an extensive in vivo investigation, specifically into the white matter connections among the hippocampal subfields and ERC in humans, considering the theorized pathways (i.e., TSP and MSP).

We extended the existing in vivo diffusion efforts (Dalton et al. 2022; Elsaid et al. 2022; Huang et al. 2021; Karat et al. 2023) to the white matter connections of the hippocampal circuit in humans by leveraging a large sample of healthy young adults. By utilizing the recent advancements in diffusion imaging, we quantified hippocampal white matter pathways in humans, including MSP‐, TSP‐related, and output connections (Figure 1b,c). This work supported the theorized anatomical structures of the hippocampal circuit derived from ex vivo and animal model studies. The individual variability we observed in the hippocampal pathways was also related to cognition. Thus, we present in vivo evidence for the feasibility of mapping the white matter connections of the hippocampal circuit in humans, using diffusion imaging. To enable further investigation of hippocampal pathways and their role in cognition, aging, and diseases, the diffusion processing pipeline and all reported data are available as a public resource with detailed documentation.

## 2 Results

### 2.1 In Vivo Reconstruction of Hippocampal White Matter Connections in Humans

**FIGURE 2:**
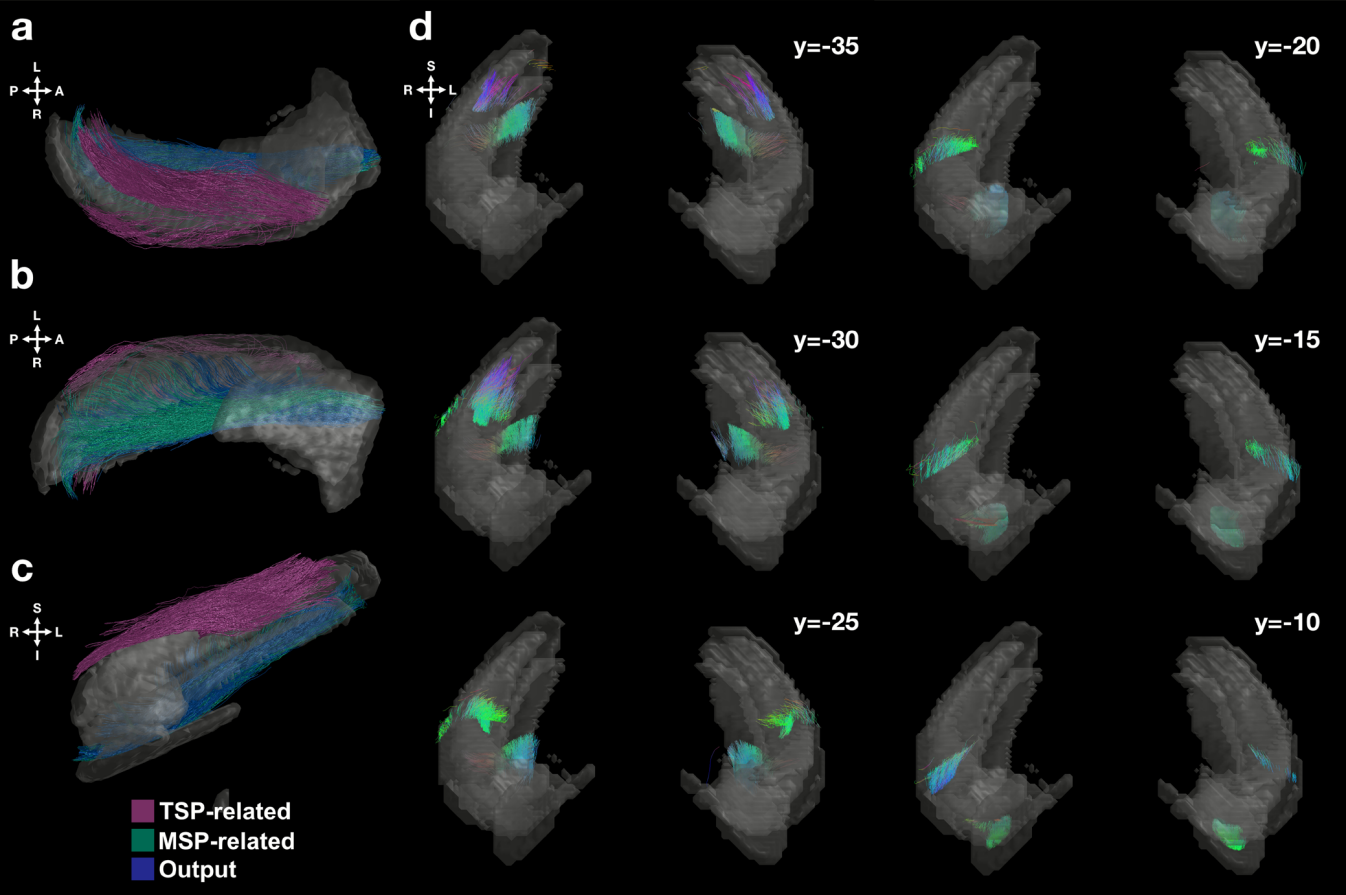
In vivo reconstruction of white matter connections in the human hippocampus. White matter connections in the human hippocampus extend from anterior to posterior end, depicted in 3D model of the hippocampus from three separate angles: (a) axial view from superior, (b) axial view from inferior, and (c) sagittal view. Right hippocampal connections are depicted as an example. These connections were based on the template image that included 653 participants and further masked by the segmentation images including the hippocampal subfields and ERC. While TSP‐related connections were more in the superior hippocampus, MSP‐related and output connections were more inferior. (d) The visualized connections are also depicted along the long axis of the hippocampus in coronal sequences. White matter connections follow the standard color‐coding of tractography: Red for right–left, green for anterior–posterior, and blue for dorsal–ventral. For oblique orientations, the colors would be blended; a combination of red and blue orientations yields magenta.

The developed pipeline demonstrated the feasibility of in vivo quantification of hippocampal white matter connections in humans with diffusion‐weighted imaging. We first generated whole‐brain tractographies at the participant level. These tractographies were then masked by the segmentation images, which included the hippocampal subfields and ERC, to isolate the white matter connections specific to the hippocampal circuit. Similar processing was also performed on the template diffusion image that was generated based on 653 participants (Figure 2a–d).

Diffusion imaging cannot reveal the directionality of the connections such as those from ERC to CA1; however, it can provide insight into the overall alignment of the connections. The reconstructed hippocampal white matter connections in humans extended along the anterior–posterior axis of the hippocampus, linking the subfields and ERC (Figure 2a–c), consistent with animal and ex vivo studies (Andersen et al. 2006; Zeineh et al. 2017) and closely aligned with earlier diffusion work in humans (Zeineh et al. 2012). TSP‐related connections fell within the more superior part of the hippocampus while MSP‐related and output connections were inferior (Figure 2a–c). Based purely on the quantified connections, hippocampal structure was readily distinguished in coronal slices, visible in the sequences of coronal sections (Figure 2d). Although most connections were in the anterior–posterior direction, certain inferior connections lay along the medial‐lateral axis of the hippocampus (Figure 2d). Additionally, toward the posterior hippocampus, some connections were positioned along the inferior–superior extent, possibly forming larger efferent white matter connections of the hippocampus (Figure 2d).

### 2.2 White Matter Connections Isolated for Specific Hippocampal Pathways in Humans

**FIGURE 3:**
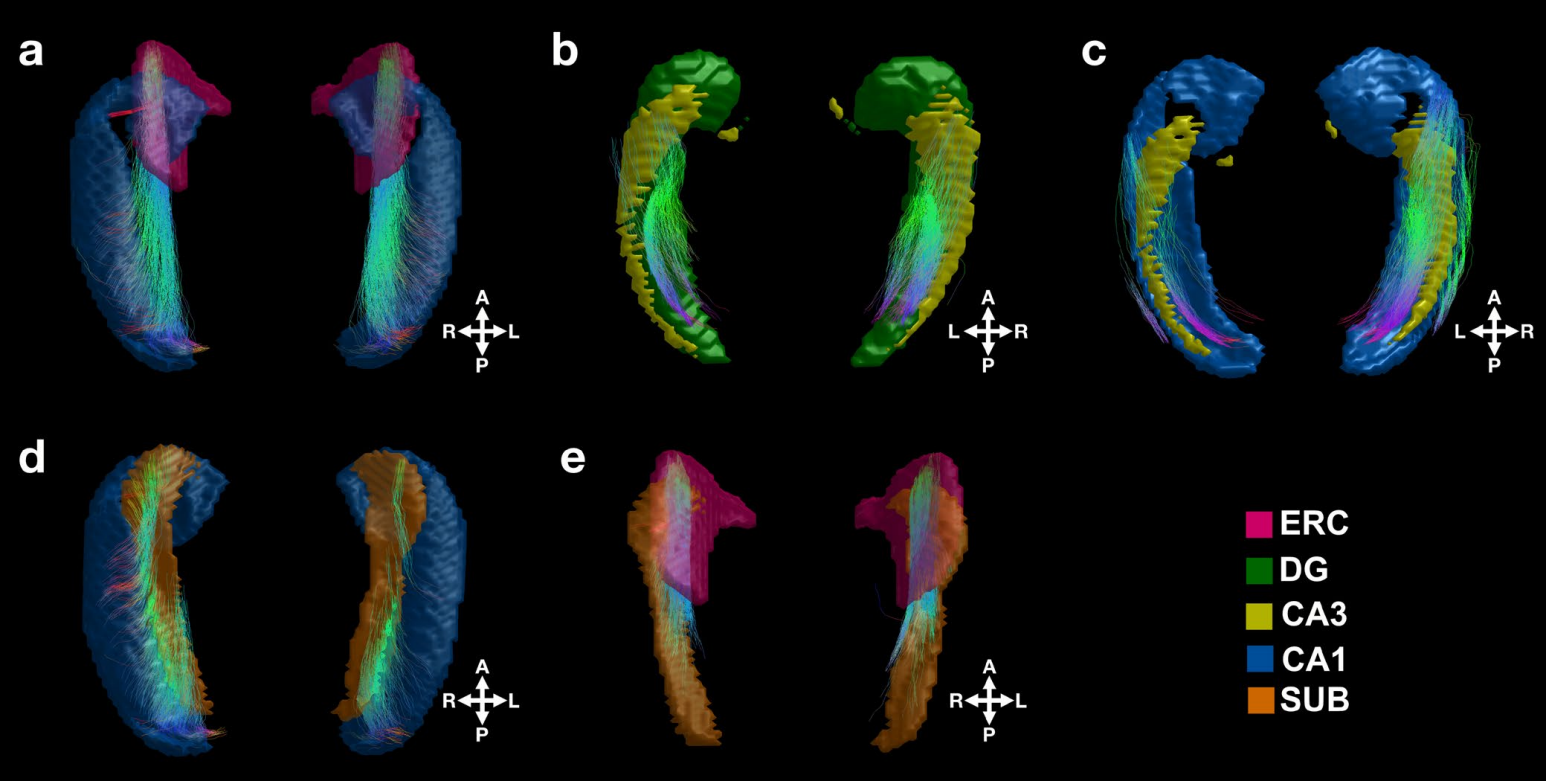
Quantified white matter connections of the theorized hippocampal pathways. The quantified human hippocampal white matter connections were isolated for traditionally defined hippocampal pathways (i.e., MSP‐, TSP‐related and output connections). The population template of hippocampal connections is depicted. (a) MSP‐related connections link ERC and CA1. (b) DG‐CA3 and (c) CA3‐CA1 connections are part of the TSP‐related connections. (d) CA1‐SUB and (e) SUB‐ERC are referred to as output connections of the hippocampal circuit.

The quantified connections in the human hippocampus were then isolated for the traditionally defined hippocampal pathways, namely MSP‐, TSP‐related, and output connections. MSP‐related connections were specific to those between ERC and CA1. These connections converged onto a relatively specific region of ERC but exhibited a wider connectivity on CA1, curving laterally toward the ends (Figure 3a). The sparse CA1 connectivity might be arising from multiple factors such as the nonuniform spatial input profile of CA1 (Druckmann et al. 2014) or scattered patterns of certain CA1 cell types (Bocchio et al. 2020).

On the other hand, TSP‐related connections presented a relatively slimmer pattern (Figure 3b,c). These included two sets of connections: DG‐CA3 (Figure 3b) and CA3‐CA1 (Figure 3c). Both TSP‐related connections (i.e., DG‐CA3 and CA3‐CA1) appeared as bundles of relatively shorter‐range connections attaching the corresponding two subfields along the long axis of the hippocampus. TSP‐related connections were positioned more superior to the hippocampus while those of MSP‐related connections were more inferior. For the rest of the analyses, TSP‐related connections were masked by relatively larger white matter structures of the hippocampus, specifically the fornix, fimbria, and mammillary bodies to avoid any potential overlap as they also lie superior to the hippocampus. Traditionally, TSP also includes connections between ERC and DG. However, these connections did not yield high‐quality reconstruction in the current work; thus, they were excluded from the rest of the analyses. The difficulty in visualizing ERC‐DG connections is due to the specific curvature of DG as reported in previous ex vivo diffusion work (Augustinack et al. 2010).

Lastly, we isolated two output connections: CA1‐SUB (Figure 3d) and SUB‐ERC (Figure 3e). We refer to these as output connections since they complete the hippocampal circuit. CA1‐SUB connections resembled MSP‐related connections, landing on a widespread region of CA1 with curved ends. Both ERC‐CA1 and CA1‐SUB presented long‐distance connections, consistent with their description in post‐mortem work (Modo et al. 2023).

### 2.3 White Matter Connections Are Differentially Distributed in the Hippocampal Circuit

**FIGURE 4:**
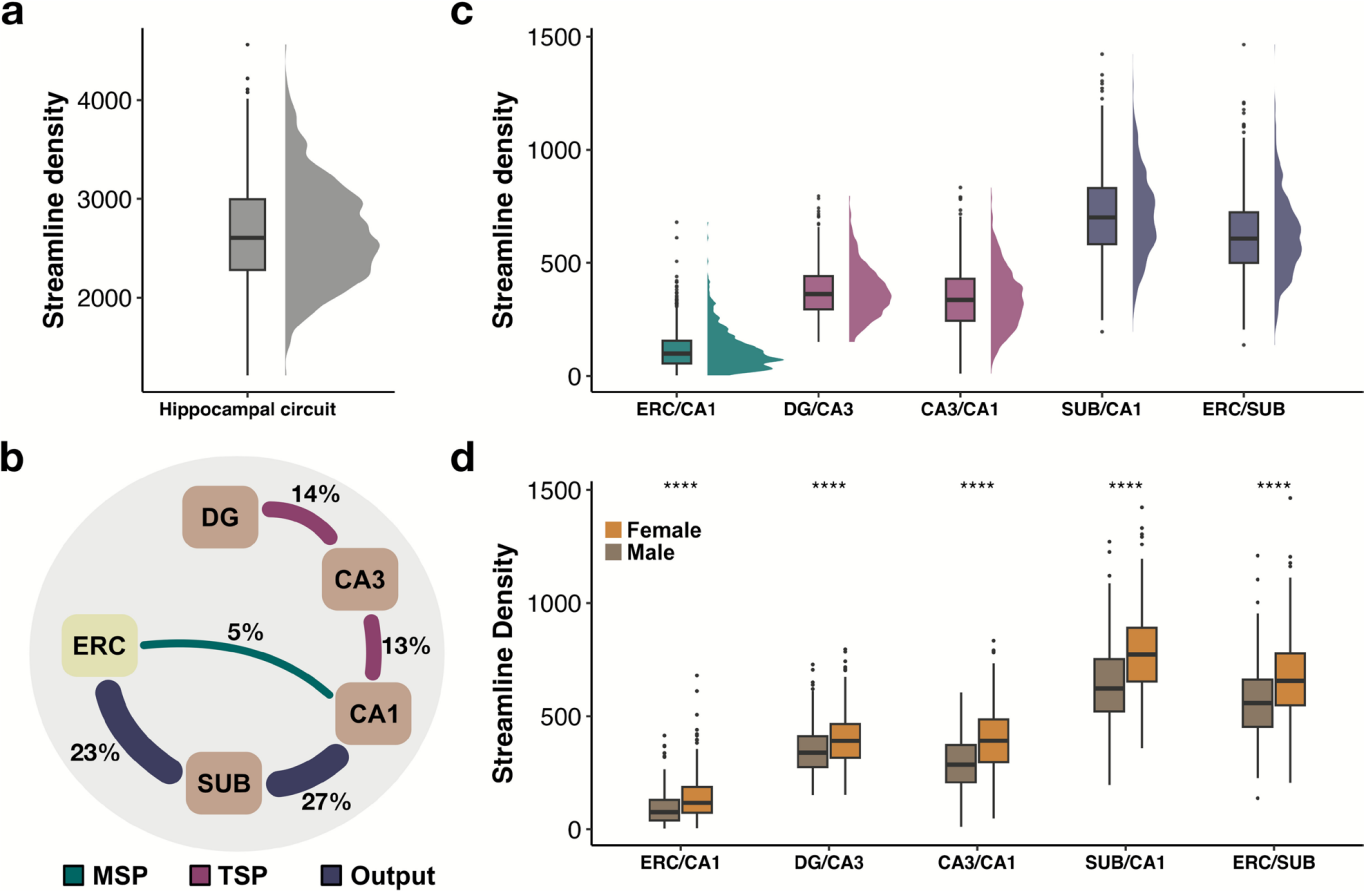
Individual variability in the differentially distributed hippocampal white matter connections. (a) Streamline density distribution in the human hippocampal circuit. (b) Hippocampal white matter connections are differentially distributed across the hippocampal pathways. Percentages represent the distribution of streamlines that belong to a particular connection between two regions. (c) Streamline density of hippocampal white matter connections, plotted across the traditionally defined hippocampal pathways. Output connections show the greatest streamline density, which is followed by TSP‐related and then MSP‐related connections. (d) Streamline density of the hippocampal pathways presents biological sex differences with greater streamline density in females than males.

Quantifying participant‐specific hippocampal white matter connections uncovered differential distribution of human hippocampal pathways. Based on 10 million streamlines in each participant, which were down sampled to 2 million for biologically plausible connections, the hippocampal circuit had a mean of 2658 ± 510 streamlines with individual differences ranging from 1213 to 4561 streamlines (Figure 4a). Averaging the streamline densities across 653 participants, we observed that the connections were differentially distributed across the traditionally defined hippocampal pathways (Figure 4b). Only about 5% of all hippocampal streamlines were within ERC‐CA1 connections, making MSP‐related connections the smallest in the hippocampal circuit. Both TSP‐related connections included twice as many streamlines as MSP‐related connections. Specifically, 14% of streamlines were part of DG‐CA3 and about 13% were within CA3‐CA1 connections, bringing TSP‐related connections to 27%. Most of the streamlines within the hippocampal circuit fell within output connections: 27% of all hippocampal streamlines were part of CA1‐SUB and 23% were within SUB‐ERC connections.

### 2.4 Individual Variability in the Human Hippocampal Pathways

In addition to assessing the distribution of streamlines across the hippocampal pathways, we evaluated participant‐specific hippocampal pathways. Analyses of variance revealed statistically significant differences between the streamline density of the five hippocampal pathways, *F*(4, 3260) = 1690, *p* = 2E−16 (Figure 4c). The streamline density of TSP‐related connections, both DG‐CA3 and CA3‐CA1, was statistically greater than MSP connections (i.e., ERC‐CA1), *p* = 2.2E−16, 95% CI = [233.86, 278.25] and *p* = 2.2E−16, 95% CI = [203.68, 248.07], respectively (Figure 4c). Within TSP‐related connections, there were significantly greater connections within DG‐CA3 than CA3‐CA1, *p* < 0.01, 95% CI = [−52.38, −7.99]. Streamline density of both output connections, CA1‐SUB and SUB‐ERC, was significantly greater than CA3‐CA1 of TSP‐related connections, *p* = 2.2E−16, 95% CI = [346.64, 391.03] and *p* = 2.2E−16, 95% CI = [253.26, 297.65], respectively (Figure 4c). Similarly, output connections, CA1‐SUB and SUB‐ERC, showed significantly larger streamline density than DG‐CA3 connections, *p* = 2.2E−16, 95% CI = [316.45, 360.85] and *p* = 2.2E−16, 95% CI = [223.08, 267.47], respectively.

The individual variability in streamline densities also differed across the five hippocampal pathways (Figure 4c, inset violin plots). MSP‐related connections exhibited very high variability in streamline density with a coefficient of variation of 74.96%, indicating a wide dispersion around the mean. Some of this variability might be driven by its skewed distribution (Figure 4c, inset violin plots). Although these connections are not zero, they are relatively small. These streamline density estimates are based on 10 million streamlines generated within the whole brain, which is a standard practice. Studies with small or medium sample sizes may benefit from the generation of more than 10 million streamlines. However, this was not computationally feasible in the current study with such a large sample size. CA3‐CA1 of TSP‐related connections presented the second greatest individual variability following MSP‐related connections with a coefficient of variation of 39.10%. Lastly, DG‐CA3 of TSP‐related connections as well as output connections, SUB‐CA1 and CA1‐ERC, included moderate variability of streamline density with coefficients of variation of 29.98%, 27.23%, and 28.59%.

### 2.5 Hemispheric Differences in the Human Hippocampal Pathways

We observed hemispheric differences in streamline density of MSP‐, TSP‐related, and output connections (Figure S1a). Pairwise comparisons revealed greater streamline density of MSP‐related connections (i.e., ERC‐CA1) in the left hippocampus than the right, *t*(652) = 2.81, *p* < 0.01 On the contrary, the rest of the hippocampal pathways presented larger streamline density in the right hippocampus than the left. Specifically, right TSP‐related connections, DG‐CA3 and CA3‐CA1, had greater streamline density than the left, *t*(652) = 3.96, *p* = 8.46E−05 and *t*(652) = 36.27, *p* < 2.2E−16. Similarly, output connections, CA1‐SUB and SUB‐ERC, in the right hippocampus included significantly larger streamline density than the left hippocampus, *t*(652) = 34.65, *p* < 2.2E−16 and *t*(652) = 10.35, *p* < 2.2E−16. All hemispheric differences remained significant after the Bonferroni correction for multiple comparisons at the alpha level of 0.01 (0.05/5 comparisons). In addition, we investigated whether the individual variability or the hemispheric difference of the hippocampal pathways was driven by unequal volumes of ERC and hippocampal subfields. We corrected the streamline density of hippocampal connections by the volume of the two regions each connection links to. Above comparisons remained significant, indicating no volume effects on our results (Figure S1b).

### 2.6 Sex Differences in the Human Hippocampal Pathways

All quantified human hippocampal pathways (i.e., MSP‐, TSP‐related, and output connections) demonstrated biological sex differences with greater streamline density in females than males (Figure 4d). Specifically, females presented significantly greater streamline density in MSP‐related connections (i.e., ERC‐CA1), *t*(625.74) = 6.81, *p* = 2.3E−11. Females also had greater streamline density in both TSP‐related connections (i.e., DG‐CA3 and CA3‐CA1), *t*(649.8) = 5.88, *p* = 6.7E−09 and *t*(635.76) = 10.80, *p* < 2.2E−16. Lastly, both output connections, CA1‐SUB and SUB‐ERC, also had significantly more streamline density in females than males, *t*(648.85) = 9.57, *p* < 2.2E−16 and *t*(645.29) = 7.76, *p* = 3.3E−14.

### 2.7 Individual Variability in TSP Connections Relates to Cognition

**FIGURE 5:**
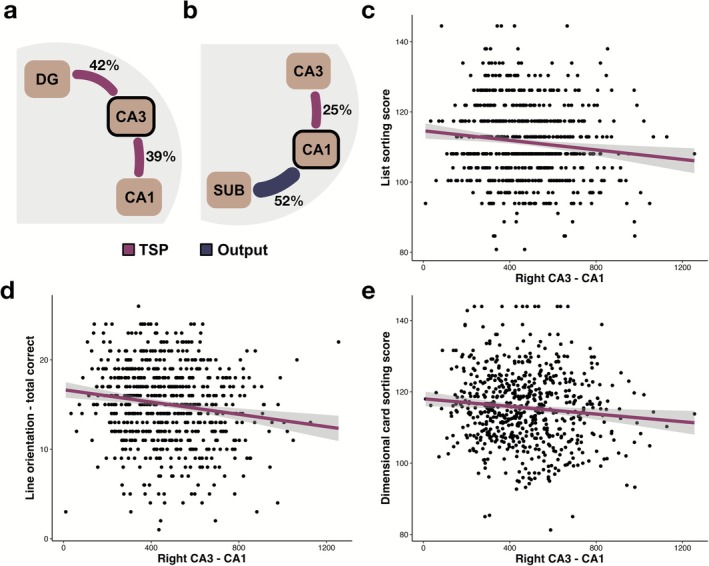
Individual variability in trisynaptic pathway‐related connections is linked to cognition. (a) CA3 is densely connected to DG and CA1 within the TSP subnetwork. (b) Most of CA1's connections are shared with SUB, but a significant percentage of its connections are linked to CA3. Individual variability in the streamline density of right CA3‐CA1 of TSP‐related connections is associated with (c) list sorting scores (i.e., working memory), (d) line orientation performance (i.e., spatial orientation), and (e) showed a trending relation with dimensional card sorting ability (i.e., cognitive flexibility).

Although the proportion of TSP‐related connections (i.e., 27%) is relatively higher than that of MSP (i.e., 5%), TSP‐related connections are pooled from two separate connections, namely DG‐CA3 and CA3‐CA1. Given the importance of TSP in cognitive processing, we further explored the connectivity structure within the TSP subnetwork by estimating the connection probabilities from the perspective of each of the TSP‐related subfields, specifically with respect to CA3 (Figure 5a) or CA1 (Figure 5b). For instance, from the perspective of CA3, we quantified the total number of connections that CA3 has with all subfields in the hippocampal circuit. To estimate the TSP subnetwork proportions from the CA3 perspective, we divided DG‐CA3 and CA3‐CA1 connections by this total number of connections of CA3 within the hippocampal circuit. Similarly, from the perspective of CA1, CA3‐CA1 and CA1‐SUB connections were divided by the total number of CA1 connections within the hippocampal circuit. When we consider all hippocampal connections that land on CA3, 42% of them were linked to DG and 39% of CA3 connections landed on CA1, highlighting dense connectivity within the TSP subnetwork (Figure 5a). Similarly, 52% of CA1 connections linked to SUB to form output connections, and about 25% of CA1 connections were shared with CA3 (Figure 5b). The relative proportion of hippocampal connections within the TSP subnetwork maps out a clear pathway consistent with the theorized structure of TSP (Andersen et al. 1971; David and Pierre 2006).

We also investigated whether streamline densities of the quantified hippocampal pathways (MSP‐, TSP‐related, and output connections) were linked to cognition. Linear mixed effects models with age and sex covariance were fit to the hippocampal pathways to predict cognitive scores. We observed that a number of cognitive domains including working memory, spatial orientation, and cognitive flexibility were related to the hippocampal pathways, specifically to TSP‐related connections (i.e., CA3‐CA1) (Figure 5c–e). The streamline density of right CA3‐CA1 connections was negatively associated with list sorting scores (i.e., working memory; *β* = −1.81, 95% CI [−2.96, −0.66], *p* = 0.002; Figure 5c). Although it is unintuitive why reduced streamline density might be l to better working memory capacity, this might relate to the importance of sparse TSP‐like representations in learning and memory processes (Knierim and Neunuebel 2016; Leutgeb et al. 2007; Niibori et al. 2012). Similarly, reduced streamline density of right CA3‐CA1 was also associated with correct total scores in a line orientation task (i.e., spatial orientation; *β* = −4.35, 95% CI [−7.24, −1.46], *p* = 0.003; Figure 5e). These results remained significant after multiple comparisons corrections with the Bonferroni test. Right CA3‐CA1 only had a trending relationship with performance in a dimensional card sorting task (i.e., cognitive flexibility; *β* = −1.52, 95% CI [−2.75, −0.28], *p* = 0.016; Figure 5d). We also observed that the streamline density of right SUB‐CA1 was associated with verbal episodic memory scores (*β* = 1.25, 95% CI [0.28, 2.22], *p* = 0.012). However, these two latter associations did not survive the Bonferroni correction at the alpha level of 0.01, resulting in only a trending significance. In our additional analyses, we ran separate linear mixed effects models that accounted for subfield volumes in addition to sex and age. The reported relationships remained the same (Table S1).

## 3 Discussion

We provide in vivo evidence for the hippocampal pathways in humans, which support the neurobiological description of the hippocampal circuit developed by animal models and theoretical work (Andersen et al. 2006; Zeineh et al. 2017). We demonstrated the interconnectedness of the hippocampal subfields and ERC in humans as white matter connections extended along the long axis of the hippocampus. The traditionally defined hippocampal pathways, including MSP‐, TSP‐related, and output connections, emerged from the quantified white matter connections, verifying the feasibility of diffusion methods for in vivo investigation of the human hippocampal circuit. We observed that human hippocampal white matter connections were differentially distributed across the hippocampal pathways; TSP‐related connections comprised more streamlines than MSP‐related connections but less than output connections. Moreover, characterizing hippocampal white matter connections at the participant level revealed the individual differences. Biological sex and hemisphere were only two sources of variability. Such diversity observed in the human hippocampal pathways was fundamentally linked to cognitive abilities. Thus, in vivo quantification of human hippocampal pathways is critical in understanding the role of the hippocampal circuit in cognition and its potential implications in aging and diseases.

Quantified white matter connections extended along the long axis of the human hippocampus, confirming the anatomy reported in ex vivo and animal studies (Andersen et al. 2006; Zeineh et al. 2017) and aligning with earlier diffusion work in humans (Zeineh et al. 2012). Sorting these white matter connections according to each pair of hippocampal subfields and ERC uncovered the traditionally defined hippocampal pathways in humans, including MSP‐, TSP‐related, and output connections. TSP‐related connections included twice as many streamlines as those of MSP‐related. This was consistent with ex vivo work; TSP consisted of dense connections while MSP connections were sparser (Johnston and Amaral 2004), a characteristic that was also implemented in the computational models of the hippocampus (Ketz et al. 2013; Schapiro et al. 2017). Despite fundamental differences between MSP and TSP, CA1 receives input from both ERC and CA3. We observed no overlap between these connections such that MSP‐related connections landed mostly on inferior CA1 while TSP‐related connections were more superior. Theoretical work suggests that both MSP and TSP feed into output connections, including CA1‐SUB and SUB‐ERC, which complete the hippocampal circuit.

A large percentage of quantified hippocampal connections was part of output connections, consistent with rodent work that CA1, SUB, and ERC are heavily interconnected (Naber et al. 2001; Tamamaki and Nojyo 1995). In addition to connections from CA1 to SUB, tracing studies in rodents revealed backward projections from SUB to CA1 (Xu et al. 2016). Future research should investigate whether the strong connections we observed between CA1 and SUB arise from this bidirectional connectivity. On the other hand, SUB‐ERC connections are considered a major output of the hippocampal circuit, allowing communication with subcortical and cortical regions (David and Pierre 2006; Witter et al. 2017). Dense SUB‐ERC connections might be necessary for this important function. Previously, strong SUB‐ERC connections were also reported across different species including rodents (Cembrowski et al. 2018), cats (Ino et al. 2001), and rabbits (Honda and Shibata 2017) as well as in ex vivo human hippocampus (Beaujoin et al. 2018). Thus, we extended ex vivo findings to in vivo work using diffusion imaging, confirming the organization and characteristics of the hippocampal circuit in humans (Augustinack et al. 2010; Beaujoin et al. 2018; Ly et al. 2020).

In vivo quantification of the human hippocampal pathways revealed great variability across individuals. Structural variance within the hippocampus is often related to aging and cognitive performance (Patel et al. 2020). We found that the hippocampal circuit presented hemispheric differences; TSP‐related and output connections included more streamlines in the right hippocampus, while MSP‐related connections were more prominent in the left hippocampus. These characteristics of the human hippocampal pathways could have implications in cognition, aging, and diseases. For instance, recent diffusion work reported reduced structural integrity in hippocampal connections in older adults, with more pronounced aging effects in the left hippocampus (Elsaid et al. 2022). Considering our results that the left hippocampus includes fewer streamlines in most pathways, the question becomes whether aging differentially influences the right versus the left hippocampal circuit. Such structural variability in the hippocampal circuit likely arises during development, shaping cognitive abilities throughout the lifespan, as white matter pathways mature at different rates from early childhood to adulthood (Lebel et al. 2019). As one specific example, the differences in episodic memory performance in early childhood can be predicted by the white matter connections between the hippocampus and parietal lobe (Ngo et al. 2017). Our processing pipeline can further our understanding of the developmental trajectories of the hippocampal pathways and their implications throughout the lifespan and in aging.

Describing individual differences of the hippocampal white matter pathways is also impactful in predicting disease outcomes. One factor that contributes to the individual differences in the human brain is biological sex. Our results revealed that females exhibited significantly greater streamline density than males. Sex is, in fact, a significant contributor to the cognitive abilities, brain structure, and the relationship between brain and cognition (Gur et al. 1999; Haier et al. 2005; Halpern 2013). Clinical work should balance sex distributions in order to avoid potential confounds. Distinct subfields of the hippocampal circuit are suggested to be differentially vulnerable to various disorders (Small et al. 2011). For example, major depression patients were reported with reduced structural connectivity within DG (Rutland et al. 2019). As a result, white matter connections between the hippocampal subfields could also be distinctly implicated in diseases. Individual differences in the hippocampal circuit can relate to the severity of disease symptomology. Understanding the variability in the hippocampal circuit, including the biological sex differences, can optimize the search for personalized treatments in diseases.

The individual differences in the human hippocampal pathways contributed to the diversity in cognitive abilities. We found that the variability in TSP‐related connections was associated with several cognitive functions including working memory, spatial orientation ability, and cognitive flexibility. With its critical role in declarative memory formation, some consider TSP as the backbone of information processing in the hippocampus (Zorumski 2011). We previously reported the importance of TSP in learning exceptional items that need to be distinguished from similar items in memory (Schlichting et al. 2021). This ability to discriminate similar experiences was reduced in older adults, which was linked to age‐related structural degradation of TSP‐like connections (Yassa et al. 2010). Although the cognitive assessments available in this current study did not relate to MSP‐like connections, in theory, various cognitive functions are differentially supported by the hippocampal pathways. In vivo quantification of the human hippocampal pathways creates an opportunity to further investigate the contributions of MSP and TSP in cognition. Indeed, computational work posits distinct functions of the hippocampal pathways: TSP rapidly binds elements of a specific experience into distinct memory traces, and MSP slowly builds unitized representations over many experiences (Schapiro et al. 2017). We introduce an analytical processing resource that can provide empirical evidence for the prominent theories.

Recent developments in diffusion techniques accelerated the in vivo characterization of hippocampal connections with subcortical and cortical regions (Dalton et al. 2022; Elsaid et al. 2022; Maller et al. 2019; Treit et al. 2018). For instance, systematic evaluation of diffusion imaging revealed that the hippocampus was much more connected to cortical regions than it was previously thought, providing the first in vivo evidence for hippocampal–cortical white matter connections in humans (Maller et al. 2019). Higher angular and diffusing sampling resolution enabled the detection of hippocampal connections with the cingulate, which was not possible in previous diffusion studies (Maller et al. 2019). It was later reinforced that the hippocampus had several direct connections with cortical regions, including strong connections with early visual cortical areas (Huang et al. 2021). In other words, the hippocampus might be communicating with cortical regions without a gateway region such as ERC. Indeed, the anterior–posterior extent of the human hippocampus is preferentially linked to cortical regions (Dalton et al. 2022). The current processing pipeline developed for the hippocampal circuit can easily be adapted to investigate the connections of the hippocampal subfields with various regions.

Human hippocampal pathways have not been extensively studied in vivo despite the advancements in neuroimaging methods. One concern in investigating these anatomical connections using diffusion imaging is the abundance of crossing fibers in the human brain. Novel processing algorithms such as Constrained Spherical Deconvolution have been proposed to overcome these limitations. This algorithm estimates a distribution of fiber orientations in each voxel in a diffusion image, unpacking the information in the voxels regarding the crossing fibers and thus yielding a much more accurate tractography (Tournier et al. 2007). Incorporating such algorithms with high‐resolution images has proven to be effective in in vivo verification of human white matter connections of small and complex structures such as the hippocampus (Elsaid et al. 2022; Huang et al. 2021; Karat et al. 2023; Maller et al. 2019; Treit et al. 2018; Zeineh et al. 2012). We leveraged a similar methodology to quantify and characterize white matter connections within the hippocampus, pioneering in vivo human investigations of the hippocampal pathways. Although the diffusion data we are utilizing is multi‐shell, the resolution of anatomical images might be a limitation. For example, we were not able to detect the white matter fibers between DG and ERC. A similar issue was encountered in a previous ex vivo diffusion tensor imaging study that visualized the perforant pathway in the human hippocampus (Augustinack et al. 2010). High‐resolution diffusion and anatomical data with faster repetition time and multi‐shell acquisitions could be implemented to overcome these limitations.

There were certain limitations within the current work. The quality of the segmentation implemented in the pipeline has a prominent effect on the resulting hippocampal pathways. It is highly recommended that high‐resolution T2‐weighted images, especially in coronal slices, are acquired for higher specificity. As these were unavailable in the current dataset, the segmentation was performed on T1‐weighted images using the Multiple Automatically Generated Templates for different Brains (MAGeT Brain) algorithm (Chakravarty et al. 2013). While utilizing the reported processing pipeline, it is critical to implement the segmentation tool that is most relevant to the specific research question. The segmentation of hippocampal subfields is especially important because anatomical definitions of these subfields vary significantly among research groups and segmentation tools (Yushkevich et al. 2015). Consequently, the reconstruction of hippocampal pathways relies on these hippocampal subfield segmentations. For instance, as part of the automated segmentation procedure within MAGeT Brain, we implemented the atlas of the Extra‐Hippocampal White Matter Atlas and Hippocampal Subfields (Amaral et al. 2018; Winterburn et al. 2013). Within this atlas, the deeper layers of the hippocampus such as the stratum radiatum/stratum lacunosum/stratum moleculare were labeled separately from the hippocampal subfields. Although such a distinction has anatomical significance, these layers span CA1, CA2, and CA3. In the current work, it was necessary to exclude these deeper layers to avoid any overlap between the segmented regions that were used to mask the whole‐brain tractographies. The definition of hippocampal subfields in this manner was a limitation as it led to relatively fewer connections in the core of the hippocampus although some posterior connections did enter relatively deeper layers of the hippocampus. While the reported hippocampal pathways appear distinct from some canonical descriptions, they closely align with previous diffusion work in humans (Zeineh et al. 2012). We report the best estimates of the hippocampal pathways afforded by this dataset and the implemented tools. Moreover, we provide a processing pipeline for the quantification of the human hippocampal circuit with specific recommendations. Other factors related to image acquisition and processing should also be taken into consideration while characterizing hippocampal white matter microstructure (Karat et al. 2024). The strength of this work lies in the processing pipeline itself, which could further be improved with newer diffusion algorithms and tools. Now that this pipeline is available to the research community with certain guidelines, better estimates of the hippocampal pathways can be achieved with higher resolution datasets.

Overall, by harnessing cutting‐edge neuro‐analytic techniques, we extended our understanding of the hippocampal pathways that are established in theoretical, ex vivo, and animal work to in vivo human investigations. We characterized MSP‐, TSP‐related, and output connections at the participant level and evaluated the individual differences in the human hippocampal circuit. Participant‐specific white matter connections provided here offer a reference for future studies on healthy young adults, relating the hippocampal pathways to cognition. This also opens the door for multimodal investigations within the Human Connectome Project with its extensive data that is well beyond what is reported here. In addition, the population‐based hippocampal circuit provides a template that future studies can leverage while reconstructing the human hippocampal pathways. Lastly, we provide a well‐documented processing pipeline that can be modified and utilized in the reconstruction and quantification of the human hippocampal pathways in various populations from clinical or aging to developmental studies. Thus, this project paves the way for understanding the hippocampal circuit in humans and its implications in cognition, aging, and diseases.

## 4 Methods

### 4.1 Human Connectome Project Participants

We analyzed diffusion and anatomical MRI volumes of 831 healthy adult participants (*F* = 445, *M* = 386; ages 22–35) from the Human Connectome Dataset, specifically the Human Connectome Project 1200 subject release (Van Essen et al. 2013). Out of 1206 subjects in this release, we included those who had at least one T1, one T2, and one diffusion‐weighted volume. Within this subset, 57 participants were reported with structural or segmentation abnormalities by the Human Connectome Project team, 65 resulted in a faulty segmentation, and 3 yielded problematic Fiber Orientation Distribution (FOD) images in the MRtrix pipeline. Additionally, participant‐specific hippocampal segmentations were further quality checked, and 178 participants were excluded as their segmentation images did not fully capture hippocampal subfields, leaving a total of 653 subjects for the central analysis. All Human Connectome Project participants provided informed consent. The study was approved by the Institutional Review Board at Washington University in St. Louis. The use of the Human Connectome Project data for the current analyses was conducted in accordance with the University of Toronto Research Ethics Board.

### 4.2 Human Connectome Project MRI Acquisition

Details related to image acquisition (Sotiropoulos et al. 2016) and preprocessing (Glasser et al. 2013) are published elsewhere. Scans were performed in a 3 T Siemens Connectome Skyra equipped with a 100 mT/m gradient set and 32‐channel receive coils (Uğurbil et al. 2013). Nominal spatial resolution was 1.25 mm isotropic (matrix size PE × Readout = 144 × 168 with left–right [LR] phase encoding [PE] and 6/8 PE partial Fourier). A total of 108 echoes are collected, with echo spacing of 0.78 ms and readout bandwidth 1490 Hz/pixel, resulting in a total echo train length (ETL) of 84.24 ms. Sampling in q‐space includes three shells at *b* = 1000, 2000, and 3000 s/mm^2^ and 18 *b* = 0 s/mm^2^ images. TE (89 ms) and TR (5.5 s) are matched across shells. Total scanning time for this protocol was about 55 min. The Human Connectome Project minimal preprocessing pipeline used on this dataset (Glasser et al. 2013) included artifact removal, motion correction, and registration to standard space. T1‐weighted structural images were acquired with a size of 0.7 mm isotropic, TR = 2400 ms, TE = 2.14 ms, flip angle = 8°, and FOV = 224 × 224 mm.

### 4.3 Hippocampal Subfield Segmentation

The MAGeT Brain algorithm (Chakravarty et al. 2013) was used to segment the hippocampus into its subfields. This segmentation was performed using the Extra‐Hippocampal White Matter Atlas and Hippocampal Subfields (Amaral et al. 2018; Winterburn et al. 2013) that generated the following subfields separately in the left and right hemispheres: CA1, SUB, CA4/dentate gyrus, CA2/CA3, as well as stratum radiatum/stratum lacunosum/stratum moleculare which was not included in the tractography.

This procedure also produced hippocampal white matter segmentation, including the fimbria, mammillary body, fornix, and alveus. White matter segmentations were used as a mask to increase the specificity of TSP‐related connections. MAGeT Brain automates a multi‐atlas‐based segmentation procedure in which a subset of participants is first registered to manually labeled atlases to generate a library of template segmentations. Each participant from the entire dataset is then registered to each of the template participants and the multiple related template segmentations are then combined via voxel‐by‐voxel label fusion to generate a final participant‐specific segmentation. For datasets with only T1 anatomical volumes, the multi‐atlas segmentation approach has been demonstrated to be more sensitive and accurate relative to other single‐atlas methods (Bussy et al. 2021; Chakravarty et al. 2013; Yushkevich et al. 2014).

### 4.4 ERC Segmentation

We segmented ERC, using the Automatic Segmentation of Hippocampal Subfields (ASHS) algorithm (Yushkevich et al. 2014) and ASHS Penn Memory Center T1‐Only Atlas (PMC‐T1) (Xie et al. 2016). This segmentation process also included the medial temporal lobe (MTL) regions (i.e., perirhinal and parahippocampal cortices along with ERC) and anterior/posterior hippocampus. ASHS first creates an atlas library of manually segmented brains each registered to a template participant. Then, labels are fused across atlases to generate the final segmentation. This procedure was completed using the Distribution Segmentation Service of ITK‐SNAP (Yushkevich et al. 2006). Within the resulting segmentation, only ERC was combined with the hippocampal subfield segmentation and used in the remaining analyses.

### 4.5 Fiber Orientation Distributions

**FIGURE 6:**
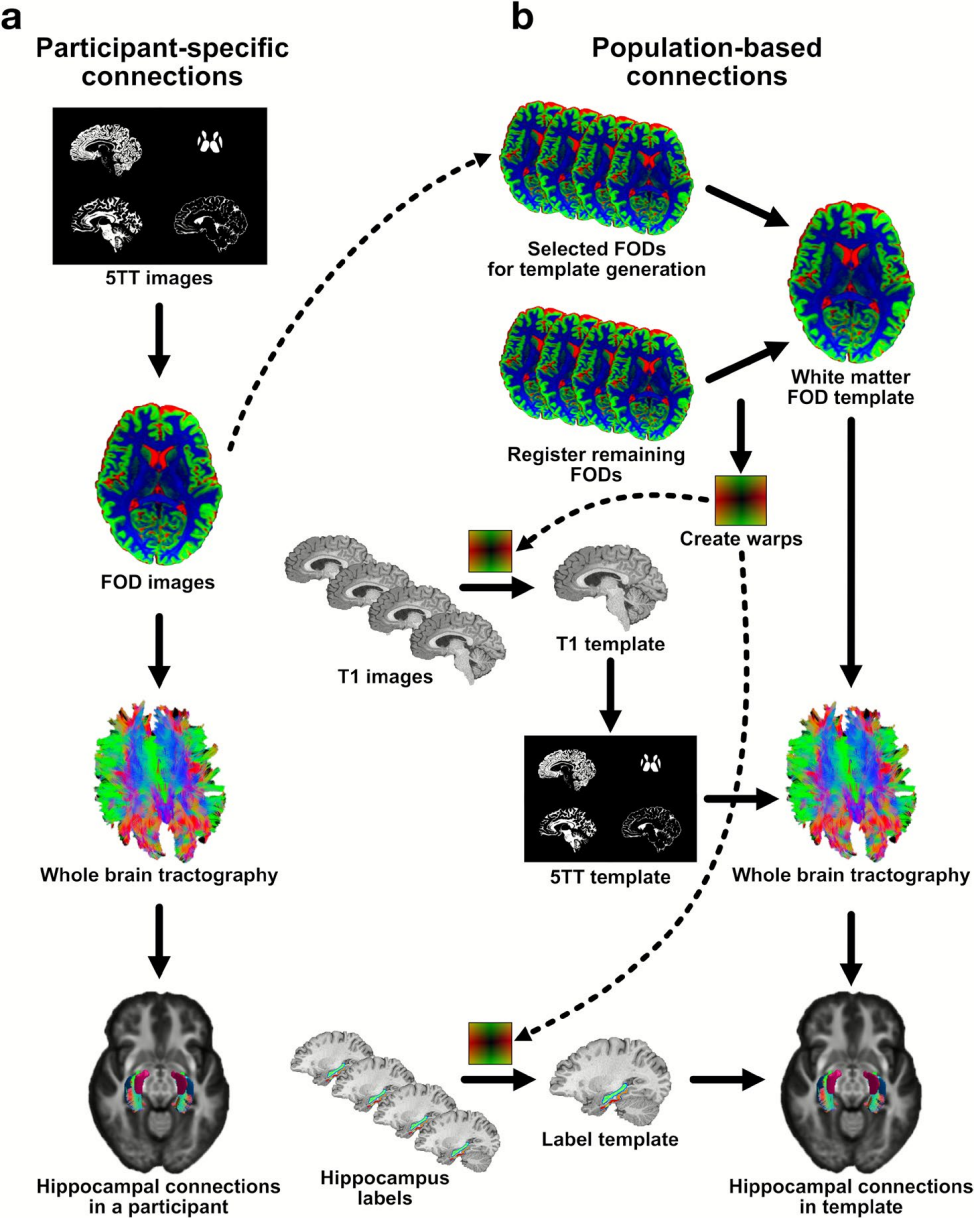
Processing pipeline for quantifying hippocampal white matter connections in humans. (a) The first arm of the processing pipeline is for reconstruction of hippocampal white matter connections at the individual level. The processing includes generation of five‐tissue type images, estimation of fiber distribution images, reconstruction of whole‐brain tractography, and creation of hippocampal connections by masking the whole‐brain tractography by the segmentation images that include hippocampal subfields and ERC. (b) The second arm of the pipeline enables the creation of hippocampal white matter connections based on the entire sample. This follows the same steps as the first arm; however, the processing here is based on the template images that are warped into the template space, not subject‐specific images.

Whole‐brain tractography and resulting connectomes for each participant were generated (Figure 6a), using MRtrix3 (Tournier et al. 2019). This tool allows for the characterization of fiber populations within each voxel (i.e., fixel) through Multi‐Shell, Multi‐Tissue Constrained Spherical Deconvolution (MSMT‐CSD) and produces a precise estimation of crossing fiber orientation (Jeurissen et al. 2014; Tournier et al. 2012). We first estimated response functions for three different tissue types (white matter/gray matter/cerebrospinal fluid) based on co‐registered five‐tissue type (5TT) images derived from participants' T1 images (*dwi2response* with *msmt_5tt* algorithm) (Jeurissen et al. 2014) (Figure 6a). We then estimated fiber orientation distribution (FOD) based on these response functions (*dwi2fod* with *msmt_csd* algorithm) (Jeurissen et al. 2014).

### 4.6 Whole‐Brain Tractography Reconstruction

Based on the white matter FOD images, whole‐brain probabilistic tractography for each participant was created using *tckgen* with the *iFOD2* algorithm and the Anatomically‐Constrained Tractography (ACT) framework (Smith et al. 2012; Tournier et al. 2019). Each tractography was generated with 10 million streamlines, using the curvature threshold of 45°, maximum fiber length of 250 mm while seeding from the gray matter–white matter interface and retracing poor structural terminations (Smith et al. 2012; Tournier et al. 2010). We then applied spherical deconvolution informed filtering to match streamline densities with the FOD lobe integrals, allowing the removal of false‐positive tracks and creating more biologically meaningful estimates (Smith et al. 2013). With this step, final whole‐brain tractography of each participant included 2 million streamlines.

### 4.7 Human Hippocampal Pathways at the Individual Level

Structural connectomes were generated based on subject‐specific whole‐brain tractography and segmentation images (hippocampal subfields + ERC) using *tck2connectome* (Tournier et al. 2019). Each resulting connectome contained the density information of streamlines between the hippocampal subfields and ERC. Each individual hippocampal connectivity matrix consisted of five regions of interest (ROIs) in each hemisphere: CA1, SUB, CA4/dentate gyrus, CA2/CA3, and ERC. Each entry in these connectivity matrices represents the streamline density between two ROIs in each participant‐specific segmentation image. Streamlines specific to the hippocampal circuit were then extracted based on these connectivity matrices, using *connectome2tck*. The resulting streamlines were carefully inspected for erroneous streamlines that extend beyond the hippocampus or ERC (Figure S2). Depending on the data implemented, streamlines can be further masked by the ROIs. In our case, we used the segmentation images (hippocampal subfields and ERC).

Based on the resulting connectomes, we quantified the streamlines for the theorized hippocampal pathways: (1) TSP‐related connections refer to DG (combined with CA4)‐CA3 (combined with CA2) and CA1‐CA3, (2) MSP‐related connections include ERC‐CA1, and (3) output connections include those between CA1‐SUB and SUB‐ERC. Importantly, seeding from the gray matter–white matter interface, combined with the ACT algorithm, was an important step in the reconstruction of streamlines within the hippocampal circuit. Generating streamlines from each voxel of the gray matter–white matter boundary respects the thin surface of the ROIs. This approach allowed for more biologically plausible connections where each connection landed on its two respective subfields. Unfortunately, we were unable to track white matter connections of ERC‐DG, which is part of TSP. The difficulty in detecting these fibers has been previously reported (Augustinack et al. 2010). DG has a deep convolution and significantly small size (Hevner 2016), which might make it difficult to identify the fibers between ERC and DG. This might also be due to the curvature of the hippocampus such that SUB is located between ERC and DG, and thus, most of the connections between ERC and DG might be instead terminating at SUB in our analyses (Augustinack et al. 2010).

Characterizing participant‐specific hippocampal pathways allowed us to estimate the proportion of connections among the hippocampal subfields and ERC, indicating the relative streamline density of such connections. The streamline density of these connections was further explored in terms of hemispheric and sex differences. We leveraged the individual variability within the hippocampal connections to explore their relation to various cognitive measures.

### 4.8 Population Template of White Matter Fiber Orientation Distributions

White matter FOD images of 30 participants, representing the available sex and age range, were selected for population template creation (Figure 6b). The remaining participants' FOD images were then registered to this white matter FOD template. Warps created during this registration were used on both individual T1 and segmentations (hippocampal subfields + ERC) to align and orient them in the population template space. Warped T1 images were averaged to create the T1 template, which was then used to create the 5TT template. A label template was generated by aggregating across participants' warped label volumes and selecting for each voxel the ROI label observed in the majority of participants. Lastly, the 5TT and white matter FOD templates were used to create the whole‐brain tractography for the population. The label template was applied to the tractography to create a population‐based connectome that was used to identify white matter streamlines among the hippocampal subfields and ERC. Streamlines between each ROI pair were edited using exclusion masks that consisted of 2.5–5 mm dilated ROI labels.

### 4.9 Quality Control

We followed a multistep quality control to ensure the accuracy of all images in each step of the processing pipeline. While implementing this pipeline, visual inspection of all images is highly recommended as there could be variations depending on the quality of diffusion and structural images or the choice of segmentation tool.

In addition to the quality control provided by the Human Connectome Project, all T1 images included in this study were visually and independently inspected by M.G. and A.B. Participants flagged with abnormalities overlapped with other exclusion criteria and were excluded. In addition, we computed the Pearson correlation between the individual T1 images and the T1 template. Approximately 96% of participants yielded a correlation coefficient equal to or larger than 0.85, showing a reliable match between individual T1 images and the template created.

The quality control of segmentation images was adapted from the protocol introduced by the Hippocampal Subfield Group (Canada et al. 2024; Canada et al. 2023). M.G. and A.B. visually inspected each participant's segmentation images (i.e., hippocampal subfields + ERC) independently before tractography reconstructions. Each segmentation image was flagged if anatomical errors were detected. The conflicts between the two reviewers were resolved by the senior author, M.L.M., which involved only a few participants. The segmentations flagged with inaccuracies were reprocessed and relabeled. The segmentation images without anatomical errors were included in the rest of the analyses. Samples of participant‐specific segmentations are depicted in Figure S3. Sixty‐five participants did not yield proper segmentations and thus were excluded from the dataset. Additionally, 178 participants yielded hippocampal segmentations that did not fully cover the subfields. Even if the segmentations were off only by a few voxels, they were excluded from the analyses, leading to *N* = 653. Subject‐specific hippocampal subfield and ERC segmentations showed a reliable overlap with the label template as 99% of participant segmentations yielded a DICE coefficient of ≥ 0.5. ROI volumes were also calculated and corrected with intracranial volume. Streamline densities of the hippocampal pathways were corrected for the ROI volumes. However, uncorrected streamline densities are reported here because volume correction did not make a significant difference on streamline distributions.

Resulting tractographies and all intermediate images were also inspected at the participant level. Specifically, the streamlines of the hippocampal circuit acquired through the pipeline should be inspected for anatomical reliability. These streamlines can be further masked by the ROIs, in our case the hippocampal subfields and ERC, to avoid those extending beyond the hippocampal circuit. In addition, the streamlines were also masked by relatively larger white matter structures surrounding the hippocampus, including the fornix, fimbria, and mammillary bodies to ensure that these were not accidentally included in the quantified connections for the hippocampal circuit.

### 4.10 Relating Human Hippocampal Pathways to Cognition

The hippocampus is involved in a variety of cognitive processes including learning, memory, and spatial navigation. We investigated the relationship between the human hippocampal pathways we quantified and the cognitive assessments available within the Human Connectome Project. We selected five tasks that relate to episodic memory, working memory, and spatial orientation, as well as card sorting (Corcoran and Upton 1993) that were shown to involve the hippocampus. Thus, we included picture sequence memory score (i.e., episodic memory), Penn word memory correct responses (i.e., verbal episodic memory), list sorting score (i.e., working memory), variable short Penn line orientation total correct (i.e., spatial orientation), and dimensional change card sorting score (i.e., cognitive flexibility). For each cognitive assessment, we built separate linear mixed effects models to estimate streamline density of participant‐specific hippocampal pathways with a fixed effect of cognitive score and a random effect of individual differences. Age and biological sex were included as covariates. We corrected for multiple comparisons using the Bonferroni test with an alpha level of 0.01 (*α* = 0.05/5 cognitive tasks).

## Author Contributions

M.G. conducted the analyses, developed the processing pipeline, and wrote the manuscript. A.B. and M.G. developed and built the website associated with the project. M.L.M. supervised the project and directed project progress. All authors edited the manuscript.

## Funding

This work was supported by the Vanier Canada Graduate Scholarship, Canadian Institutes of Health Research (Grant No. PJT‐178337), Brain Canada Foundation Grant, and Natural Sciences and Engineering Research Council of Canada (Grant Nos. RGPIN‐2017‐06753, RGPIN‐2024‐0588).

## Conflicts of Interest

The authors declare no conflicts of interest.

## Supporting information


**Figure S1:** Hemispheric differences in the streamline density of quantified human hippocampal pathways. (a) MSP‐related connections present greater streamline density in the left hippocampus, while TSP‐related and output connections are more prominent in the right hemisphere. (b) The streamline density of each hippocampal pathway is corrected by the average volume of the two regions it connects. Volume differences across the subfields and ERC did not influence the relative streamline densities. *Note:* *p < 0.01, ~*p* = 0.06.

**Figure S2:** Streamlines of the hippocampal circuit obtained from the processing pipeline may require further masking. (a) The resulting streamlines should be carefully inspected for their anatomical accuracy. The arrows depict the erroneous streamlines that extend beyond the hippocampus, including those extending into other subcortical structures or brainstem. These streamlines can be further cleaned through additional masking with the segmentation images. For the resulting streamlines following this final quality‐check, please see Figure 2.

**Figure S3:** Segmentation images of sample participants. (a) Each participant‐specific segmentation image was visually inspected by MG and AB. 15 randomly selected participants are depicted as examples. These segmentation images include hippocampal subfields and ERC. Deeper layers (i.e., stratum radiatum/stratum lacunosum/stratum moleculare), depicted in turquoise, were included in the quality check step but excluded from the rest of the analyses.

**Table S1:** The relationship between the trisynaptic subnetwork and cognitive scores remained significant after subfield volumes included as covariates.

## Data Availability

The code and data that support the findings of this study are openly available on Open Science Framework (OSF) at https://osf.io/9a2u4/.
