# Supplementary material for: In Vivo Quantification of White Matter Pathways in the Human Hippocampus
Source: Hum Brain Mapp. 2025 Nov 24;46(17):e70417. doi: 10.1002/hbm.70417 (PMC12644930; doi:10.1002/hbm.70417)
Supplement: Supplementary file 1 — Figure S1: Hemispheric differences in the streamline density of quantified human hippocampal pathways. (a) MSP‐related connections present greater streamline density in the left hippocampus, while TSP‐related and output connections are more prominent in the right hemisphere. (b) The streamline density of each hippocampal pathway is corrected by the average volume of the two regions it connects. Volume differences across the subfields and ERC did not influence the relative streamline densities. Note: *p < 0.01, ~p = 0.06. [file HBM-46-e70417-s001.pdf]

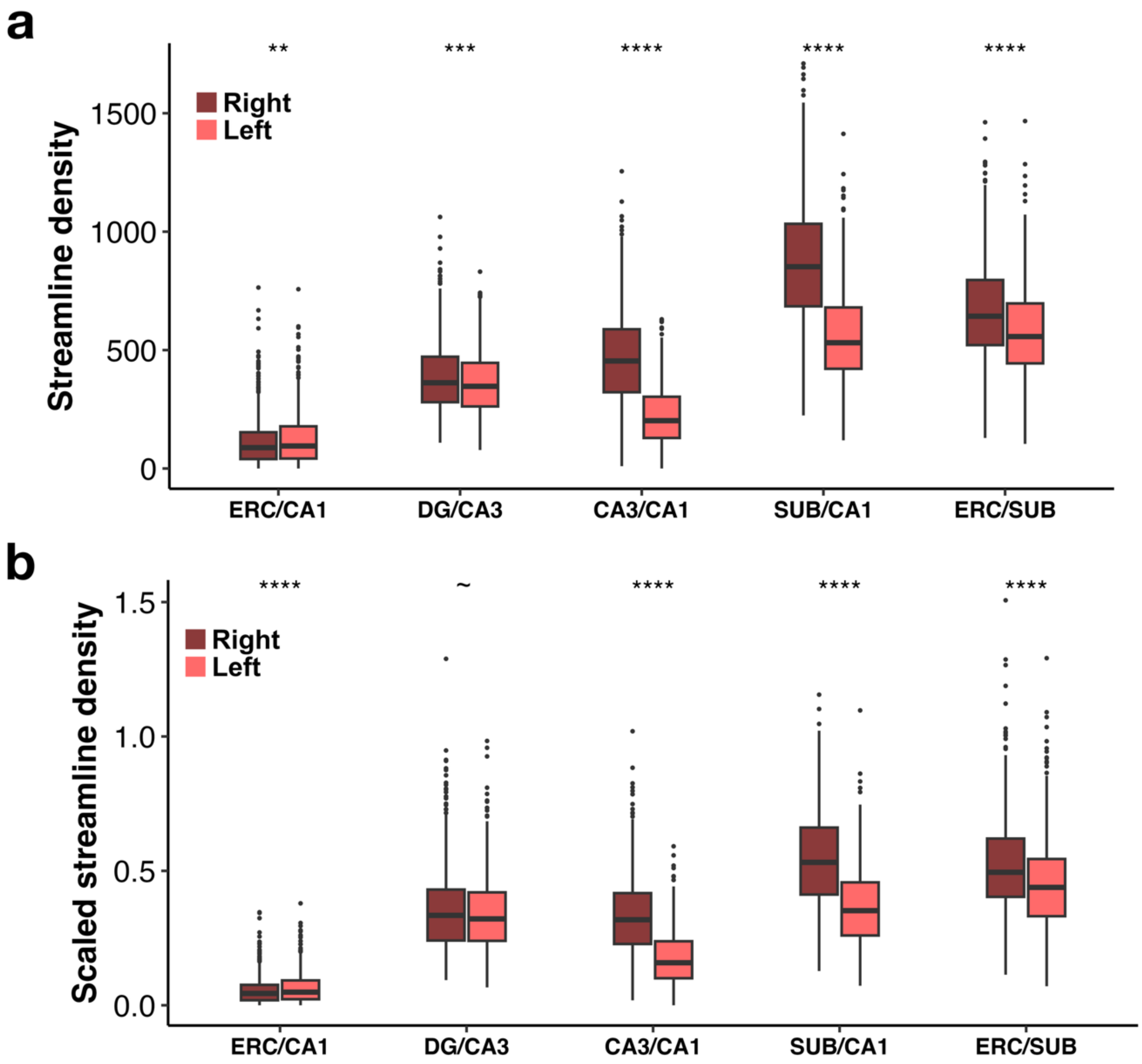

Extended Data Fig. 1: Hemispheric differences in the streamline density of quantified human hippocampal pathways. a, MSP-related connections present greater streamline density in the left hippocampus, while TSP-related and output connections are more prominent in the right hemisphere. **b,** The streamline density of each hippocampal pathway is corrected by the average volume of the two regions it connects. Volume differences across the subfields and ERC did not influence the relative streamline densities.

*Note. * p<.01, ~ p=.06*
