# Supplementary material for: In Vivo Quantification of White Matter Pathways in the Human Hippocampus
Source: Hum Brain Mapp. 2025 Nov 24;46(17):e70417. doi: 10.1002/hbm.70417 (PMC12644930; doi:10.1002/hbm.70417)
Supplement: Supplementary file 2 — Figure S2: Streamlines of the hippocampal circuit obtained from the processing pipeline may require further masking. (a) The resulting streamlines should be carefully inspected for their anatomical accuracy. The arrows depict the erroneous streamlines that extend beyond the hippocampus, including those extending into other subcortical structures or brainstem. These streamlines can be further cleaned through additional masking with the segmentation images. For the resulting streamlines following this final quality‐check, please see Figure 2. [file HBM-46-e70417-s003.pdf]

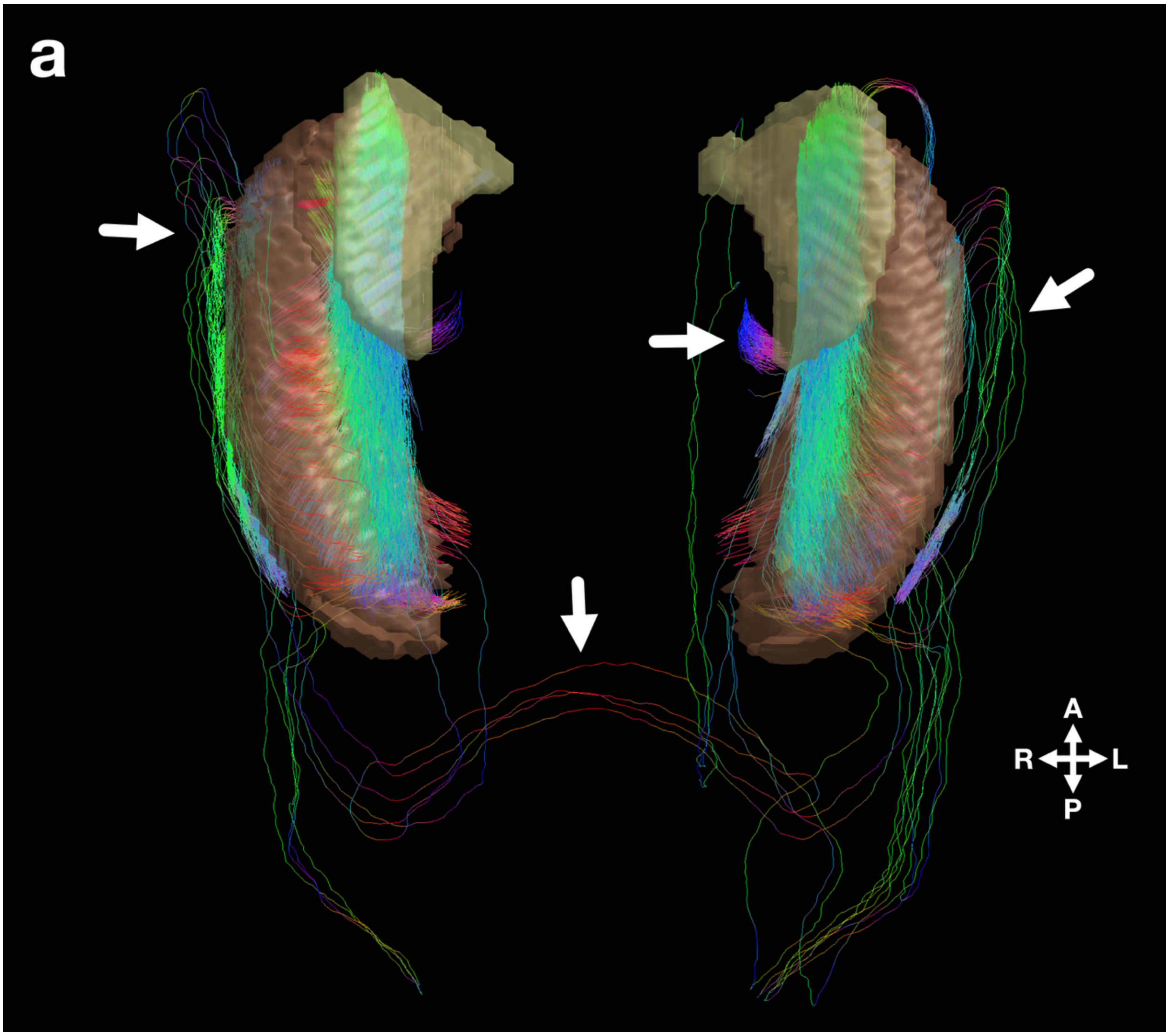

Extended Data Fig. 2: Streamlines of the hippocampal circuit obtained from the processing pipeline may require further masking. a, The resulting streamlines should be carefully inspected for their anatomical accuracy. The arrows depict the erroneous streamlines that extend beyond the hippocampus, including those extending into other subcortical structures or brainstem. These streamlines can be further cleaned through additional masking with the segmentation images. For the resulting streamlines following this final quality-check, please see Fig 2.
