# Supplementary material for: In Vivo Quantification of White Matter Pathways in the Human Hippocampus
Source: Hum Brain Mapp. 2025 Nov 24;46(17):e70417. doi: 10.1002/hbm.70417 (PMC12644930; doi:10.1002/hbm.70417)
Supplement: Supplementary file 3 — Figure S3: Segmentation images of sample participants. (a) Each participant‐specific segmentation image was visually inspected by MG and AB. 15 randomly selected participants are depicted as examples. These segmentation images include hippocampal subfields and ERC. Deeper layers (i.e., stratum radiatum/stratum lacunosum/stratum moleculare), depicted in turquoise, were included in the quality check step but excluded from the rest of the analyses. [file HBM-46-e70417-s002.pdf]

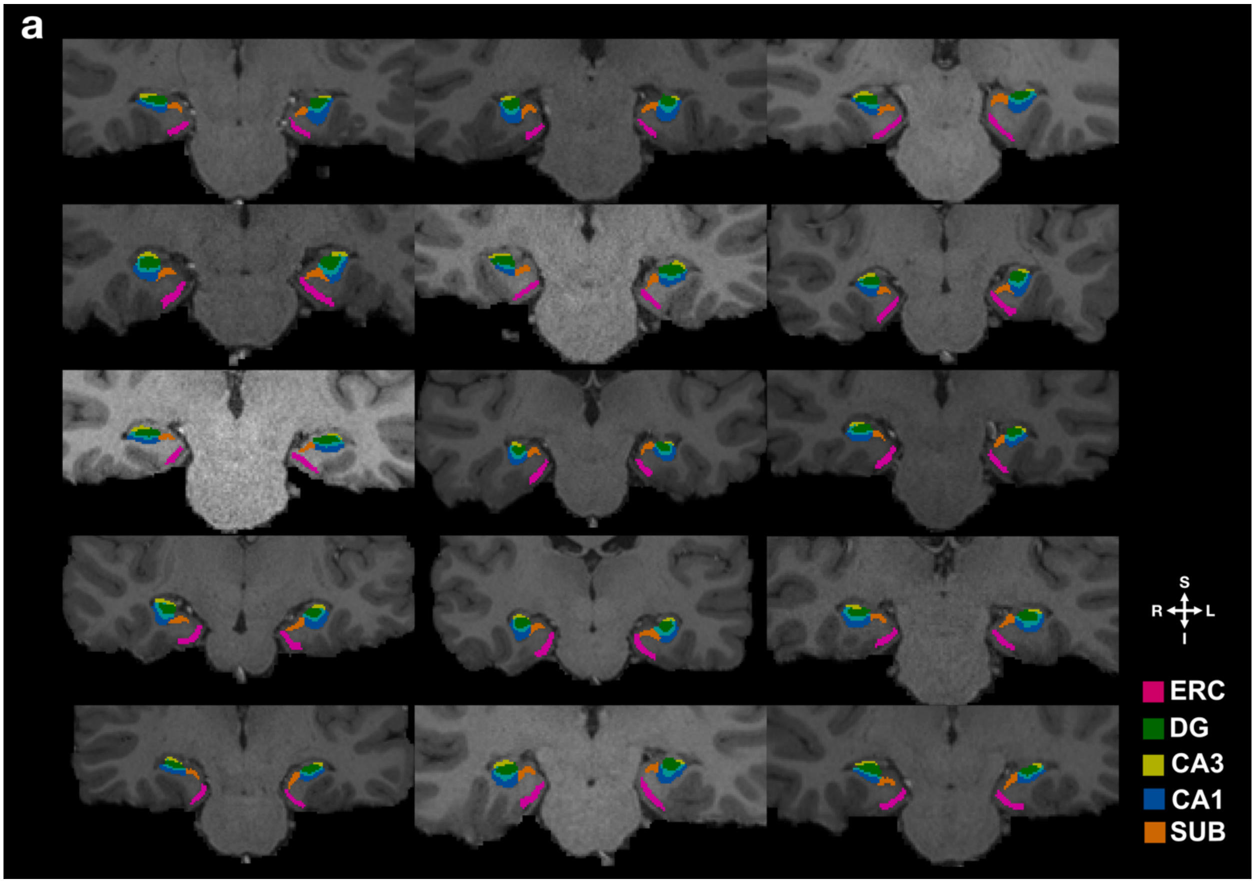


**Extended Data Fig. 3: Segmentation images of sample participants. a,** Each participant specific segmentation image was visually inspected by MG and AB. 15 randomly selected participants are depicted as examples. These segmentation images include hippocampal subfields and ERC. Deeper layers (i.e., stratum radiatum/stratum lacunosum/stratum moleculare), depicted in turquoise, were included in the quality check step but excluded from the rest of the analyses.
