# Supplementary material for: In Vivo Quantification of White Matter Pathways in the Human Hippocampus
Source: Hum Brain Mapp. 2025 Nov 24;46(17):e70417. doi: 10.1002/hbm.70417 (PMC12644930; doi:10.1002/hbm.70417)
Supplement: Supplementary file 4 — Table S1: The relationship between the trisynaptic subnetwork and cognitive scores remained significant after subfield volumes included as covariates. [file HBM-46-e70417-s004.pdf]

Extended Data Table. 1: The relationship between the trisynaptic subnetwork and cognitive scores remained significant after subfield volumes included as covariates.

Connections	Cognition	Estimates	CI	p
Right CA3-CA1	List sorting score	-1.92	[-3.06, -0.78]	<0.001*
Right CA3-CA1	Dimensional card sorting score	-1.37	[-2.60, -0.14]	0.03
Right CA3-CA1	Line orientation total correct	-5.11	[-7.99, -2.23]	<0.001*
Right SUB-CA1	Verbal episodic memory scores	1.21	[0.24, 2.18]	0.014

*Note. * Significant after Bonferroni correction at the alpha level of 0.01 (α = 0.05/5 cognitive tasks tested)*
